# Baseline heavy metals in plant species from some industrial and rural areas: Carcinogenic and non-carcinogenic risk assessment

**DOI:** 10.1016/j.mex.2018.01.003

**Published:** 2018-01-31

**Authors:** Ghasem Zolfaghari, Zohreh Akhgari Sang Atash, Ameneh Sazgar

**Affiliations:** a Department of Environmental Sciences and Engineering, Faculty of Environmental Sciences, Hakim Sabzevari University, Razavi Khorasan, Sabzevar, P.O. Box: 397, Iran; b Department of Environmental Sciences and Engineering, Gorgan University of Agriculture and Natural Resources, Gorgan, Iran; c Department of Environmental Sciences and Engineering, Ferdowsi University of Mashhad, Mashhad, Iran

**Keywords:** Heavy metals, Plant species, Industrial area, Rrural area, Risk assessment

## Abstract

This paper provides the first quantitative information on accumulation of cadmium, lead, and arsenic in the soil, leaf, and root of wheat (*Triticum aestivum* L.)*,* corn *(Zea Maize*), and tomato *(Solanum lycopersicum*) in the downstream agricultural lands of an industrial area and agricultural lands of a rural area, Razavi Khorasan province, Iran. The results showed that there is a significant difference among the cadmium concentrations in the soil, root and leaf/seed in various plants (*p* = 0.00 for wheat and corn and *p* = 0.0004 for tomato). There was no significant difference between the lead concentrations in the soil, root and leaf/seed in the case of wheat (*p >* 0.05), but there was a significant difference for other plants. Furthermore, statistical analysis was done on arsenic concentrations of soil, root and leaf/seed in the wheat, tomato, and corn. In the case of rural area, the results showed significant difference between the cadmium and lead concentrations of soil, root and leaf/seed in the wheat and tomato. In this area, statistical analysis was done on the arsenic concentrations of soil, root and leaf/seed in wheat, tomato, and corn (*p* = 0.00 for wheat, *p* = 0.00 for tomato, and *p >* *0.05* for corn). The heavy metals concentrations in some parts of the plants in the industrial area were above the standards. The concentrations of cadmium, lead, and arsenic for soil were below the limits proposed by WHO, EPA, and EU. In this study, the Total Hazard Quotient (THQ) through consumption of wheat was less than 1, indicating no significant potential health risk associated with the consumption of wheat from the areas. The cancer risk of arsenic from wheat consumption was as 255 × 10^−6^ and 0.00 in the industrial and rural areas, respectively.

• Atomic absorption spectrophotometer (AAS) equipped with graphite furnace was used for heavy metal analysis.
• The general results revealed that the levels of Cd, Pb, and As in the industrial area were higher than the rural area.
• The cancer risk of arsenic in wheat for the industrial area is greater than 1 × 10^−6^, which is unacceptable.

## Graphical abstract

**Figure:**
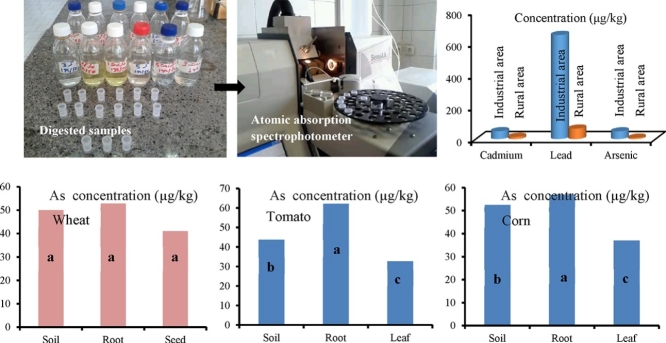

## Method details

Method name: Atomic absorption spectrophotometry and health risk assessment by EPA/WHO method

### Description of area

**Fig. 1:**
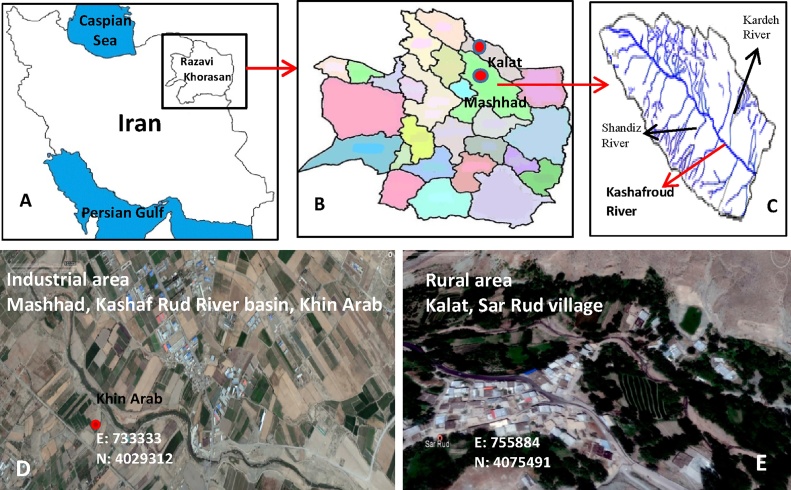
Situation of sampling sites A) Map of Iran, B) Map of Razavi Khorasan province, C) Kashaf Rud River catchment basin, D) Downstream agricultural lands of industrial area, Razavi Khorasan province, Mashhad city, Khin Arab area, Kashaf Rud River Basin, and E) Agricultural lands of rural area: Razavi Khorasan province, Kalat city, Sar Rud village.

Kashaf Rud River is located in the Eastern North of Iran between Hezarmasjed and Binaloud mountains along the west north to east south among Mashhad-Chenaran Plain. Then, it joins Hari Rud River in Sarakhs City. From that point on, it is called Tajan River, which goes to Turkmenistan. Among the most important rivers leading to Kashf Rud near Mashhad plain, we can name Ferizi, Golmakan, Shandiz, Torogh, Ardak, and Kardeh. So far, five dams of Kardeh, Torogh, Ardak, Dolatabad and Chali Darreh have been established. The water of these Streams does not arrive to the main River of Kashf Rud because of high agricultural farms and also due to its penetration into the underground water table. Kashaf Rud, today, is polluted heavily due to an entrance of industrial wastewater and agricultural runoff in Mashhad city. The agricultural land of this area is irrigated by the water of this polluted river. Sampling was done from agricultural lands of Khin Arab area located at the margin of Kashaf Rud. The second area for sampling was Sar Rud. Sar Rud is a village in Zavin Rural District, Zavin District, Kalat city, Razavi Khorasan Province, Iran. At the 2006 census, its population accounted for 849 people, in 207 families. Sar Rud is also the name of a River in this area (Sar Rud River). The agricultural land of this area is irrigated by the water of this clean river (Fig. 1).

### Sampling

**Fig. 2:**
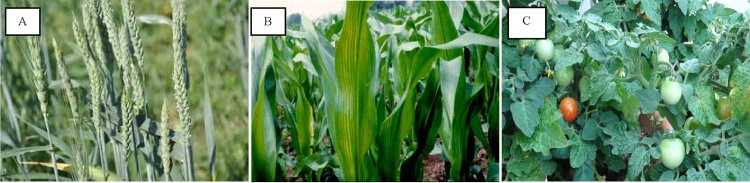
Image of plants that are used in this study. A) Wheat (*Triticu* *m aestivum L.),* B) corn (*Zea mize*) and C) tomato (*Solanum lycopersicum*) from downstream agricultural lands of industrial area, Razavi Khorasan province, Mashhad city, Khin Arab area, Kashaf Rud River Basin, and agricultural lands of rural area: Razavi Khorasan province, Kalat city, Sar Rud village.

In this study, a random sampling of soil, root and leaf/seed of wheat (*Triticum aestivum* L.), corn (*Zea mize*), and tomato (*Solanum lycopersicum*) was performed (Fig. 2). A total of 15 samples of winter wheat (from 3 farms) and some soil samples were taken and stored in the plastic containers. For corn field, which is located near to the wheat farms, in the same way, 7 samples (from two farms) were randomly selected and harvested. Samples of the two tomato farms (15 samples), approximately one kilometer from the site of the previous sampling, were also collected. A total of 111 samples for the industrial area (3 elements = 313 analysis) and 111 samples (3 elements = 313 analysis) for the rural area (soil, root, and leaf/seed) were randomly collected. After sampling, the roots and leaves were separated and dried in the open air away from direct sunlight. In the case of wheat, instead of leaves, the seeds were harvested and analyzed.

### Instrumentation

Soil samples were totally milled; then, approximately 1.5 gr of soil was placed in a furnace at a temperature of 600 °C. One gram of sample was digested in 20 ml of acid solution. Acid solution of hydrochloric acid (14 M or 37%) and nitric acid (12 M or %65) were produced at a ratio of three to one and soil samples were digested with this solution. Roots and leaves were rinsed with water, then dried and powdered. Finally, the samples were turned into ash in the oven at 600 °C and digested with nitric acid. Standards for lead analysis (50, 100 and 150 μg/L) are made of lead nitrate solution. The control solution of distilled water was considered. Standards for the analysis of cadmium and arsenic were 10, 25 and 50 μg/L. Atomic absorption spectrophotometer (AAS) equipped with graphite furnace (GBC GF3000 model) was used for heavy metal analysis [1]. A volume of 20 microliters of the sample was injected into the device.

### Statistical analysis

In this study, the SPSS software was used for statistical analysis. The data normality was done by using Shapiro-Wilk test, and the significant level of *p >* 0.05 normality was considered as a benchmark. To study the homogeneity of variances, the Leven test was used and *p >* 0.05 was considered as a benchmark. In the case of normal data and homogeneous parametric tests, Analysis of Variance (ANOVA) and Tukey test were used. Nonparametric tests, and specifically, the Kruskal-Wallis test were used for non-normal data. The level of significance was considered less than 0.05.

### Health risk assessment by EPA/WHO method

The purpose of this section was to estimate the health risks of lead, cadmium, and arsenic consumption of wheat in Mashhad provided by the US Environmental Protection Agency [2] The US EPA established the Reference Dose (RfD, μg/kg/day) as Eq. (1):

(1) $$RfD=\frac{\text{NOAEL}\;\text{or}\;\text{LOAEL}}{\text{UF}\times \text{MF}}$$

where NOAEL = No Observed Adverse Effect Level, LOAEL = Low Observed Adverse Effect Level, UF = Uncertainly Factor, and MF = Modifying Factor. Furthermore, Average Daily Dose (ADD_pot_, μg/kg/day) for exposure assessment is calculated as Eq. (2):

(2) $${\text{ADD}}_{\text{pot}}=\frac{\left(\text{C}\times \text{IR}\times \text{ED}\right)}{\text{BW}\times \text{AT}}$$

where C (μg/kg) = Concentration of toxic material, IR = Ingestion Rate (for wheat = 0.3 kg/day), ED = Exposure Duration, BW = Body Weight (70 kg), and AT = Averaging Time. The LADD (only for arsenic in wheat) takes the form of Eq. (3), with Life Time (LT) replacing the averaging time:

(3) $${\text{LADD}}_{\text{pot}}=\frac{\left(\text{C}\times \text{IR}\times \text{ED}\right)}{\text{BW}\times \text{LT}}$$

Finally for non-carcinogenic risk calculation, Total Hazard Quotient (THQ) is calculated as Eq. (3):

(4) $$\text{THQ}=\frac{{\text{ADD}}_{\text{pot}}}{\text{RfD}}$$

If the result of this formula is less than one, it indicates that the use of harmful effects on health is acute (If ADD_pot_ < RfD, then there is no problem). For carcinogenic chemicals:

(5) Cancer risk = LADD_pot_ × Slope factor

The cancer risk greater than 1 × 10^−6^ is unacceptable (usually).

## Heavy metal concentrations

**Fig. 3:**
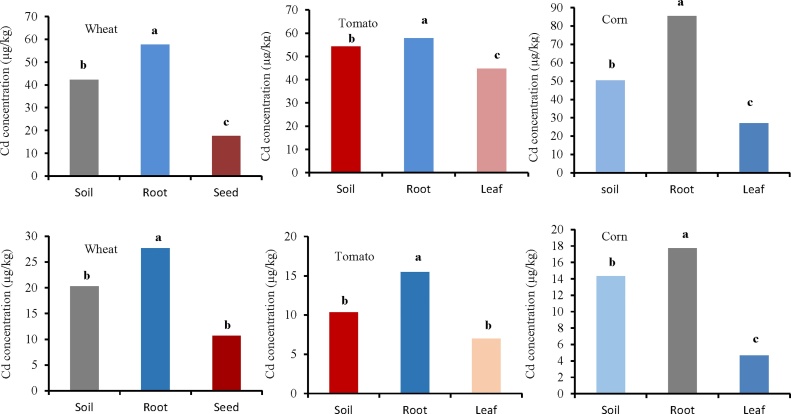
Comparison of cadmium concentration (μg/kg) in soil, root, and leaf/seed for wheat, tomato, and corn. Top three figure show plant species irrigated with wastewater (industrial area) and down three figure show plant species irrigated with river water (rural area).

The Shapiro-Wilk test showed that the Cd concentrations of soil, root, and seed for wheat in the industrial area are normal (*p >* 0.05). Furthermore, the Leven test determined that the variance among the groups are homogenous (*p *= 0.23). ANOVA test results showed a significant difference among the groups in the soil, root, and seed for wheat (*p *= 0.00) (Fig. 3 and Table 1). The results determined that the highest concentration of cadmium was in the root (57.65 μg/kg). The Shapiro-Wilk test also showed that the soil, root, and leaf data for tomato are normal (*p >* 0.05). Furthermore, the variance between the groups are homogenous (*p *= 0.82). The results showed significant difference among cadmium concentrations of soil, root, and leaf for tomato (*p *= 0.004). The highest concentration of cadmium is related to root (57.75 μg/kg). There was a significant difference among the soil, root, and leaf of corn in cadmium concentrations (*p* = 0.00). In the case of rural area, the results showed significant differences between cadmium concentrations of soil, root and leaf for wheat (*p *= 0.027), tomato (*p *= 0.027), and corn (*p *= 0.00) (Fig. 3 and Table 1).

**Table 1:** *p* value of ANOVA and Kruskal-Wallis tests for the difference in cadmium concentration (μg/kg) among soil, root, and leaf/seed (wheat, tomato, and corn) in industrial and rural areas.

Industrial area	Wheat (ANOVA (Tukey))			Tomato (ANOVA (Tukey))			Corn (ANOVA (Tukey))		
	Groups	*P*	*F*	Groups	*P*	*F*	Groups	*P*	*F*
	Soil-Root	0.008	189.17	Soil-Root	0.066	15.47	Soil-Root	0.000	78.29
	Soil-Seed	0.001		Soil-Leaf	0.077		Soil-Leaf	0.001	
	Seed-Root	0.000		Leaf-Root	0.003		Leaf-Root	0.000	

Rural area	Wheat (Kruskal-Wallis)			Tomato (Kruskal-Wallis)			Corn (ANOVA (Tukey))		
	Groups	*p*	*Z*	Groups	*p*	*Z*	Groups	*p*	*F*
	Soil-Root	0.046	−1.99	Soil-Root	0.046	−1.99	Soil-Root	0.000	685.21
	Soil-Seed	0.046	−1.99	Soil-Leaf	0.046	−1.99	Soil-Leaf	0.000	
	Seed-Root	0.050	−1.96	Leaf-Root	0.050	−1.96	Leaf-Root	0.000	

**Fig. 4:**
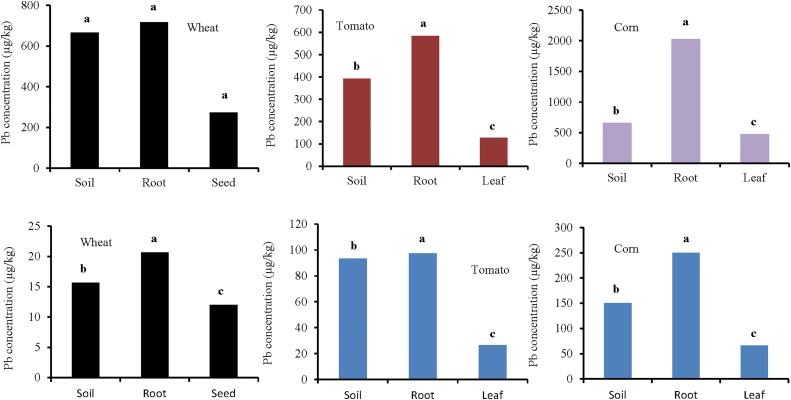
Comparison of lead concentration (μg/kg) in soil, root, and leaf/seed for wheat, tomato, and corn. Top three figure show plant species irrigated with wastewater (industrial area) and down three figure show plant species irrigated with river water (rural area).

Shapiro-Wilk test showed that the Pb concentrations of soil, root, and seed for wheat are normal (*p >* 0.05) and the Leven test revealed that the variance between the groups is not homogeneous (*p *= 0.01). Thus, the Kruskal-Wallis test showed no significant difference between the soil, root, and seed (Fig. 4 and Table 2). In addition, the results showed that the tomatoes data are normal (*p >* 0.05). The ANOVA test (Tukey) results showed a significant difference between the soil, root, and leaf for tomato (*p *= 0.00) (Fig. 4 and Table 2). Furthermore, the Shapiro-Wilk test showed that the soil, roots, and leaf data for corn are normal (*p >* 0.05). The Tukey test showed significant differences among lead concentrations in the soil, root, and leaf of corn (*p *= 0.00). In the case of rural area, the results showed significant differences among lead concentrations of soil, root, and leaf/seed for wheat (*p *= 0.00), tomato (*p *= 0.00), and corn (*p *= 0.00) (Fig. 4 and Table 2). The mean concentrations of lead for wheat in the rural area (soil = 15.66, root = 20.66, and seed = 12.00 μg/kg) indicate that the maximum concentration is in the root. Wieczorek et al. [3] reported that the cadmium concentration in the cereal grain was in all cases higher than in the soil samples. The results of several studies indicate that cadmium taken up by plants from the soil is accumulated first in roots, and then transported in smaller quantities to the stems and seeds. Cadmium concentration in other plant organs depends on numerous factors, such as specific characters of the plant species and physicochemical properties of the soil, as well as dust deposition [4]

**Table 2:** The result of Kruskal-Wallis and ANOVA (Tukey) tests for the difference in lead concentration (μg/kg) among soil, root, and leaf/seed (wheat, tomato, and corn) in industrial and rural areas.

Industrial area	Wheat (Kruskal-Wallis)			Tomato (ANOVA (Tukey))			Corn (ANOVA (Tukey))		
	Groups	*p*	*Z*	Groups	*p*	*F*	Groups	*p*	*F*
	Soil-Root	0.513	−0.65	Soil-Root	0.013	15.62	Soil-Root	0.000	64.97
	Soil-Seed	0.301	−1.96	Soil-Leaf	0.002		Soil-Leaf	0.464	
	Seed-Root	0.513	−0.65	Leaf-Root	0.000		Leaf-Root	0.000	

Rural area	Wheat (Kruskal-Wallis)			Tomato (ANOVA (Tukey))			Corn (ANOVA (Tukey))		
	Groups	*p*	*F*	Groups	*p*	*F*	Groups	*p*	*F*
	Soil-Root	0.000	100.306	Soil-Root	0.002	7.07	Soil-Root	0.001	118.35
	Soil-Seed	0.002		Soil-Leaf	0.000		Soil-Leaf	0.000	
	Seed-Root	0.000		Leaf-Root	0.000		Leaf-Root	0.000	

**Fig. 5:**
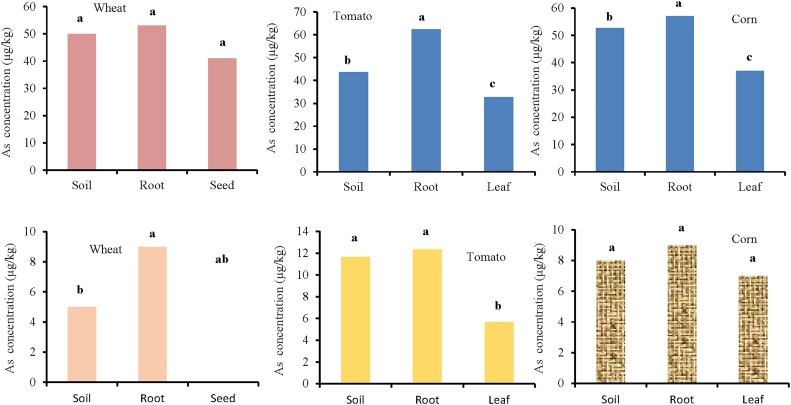
Comparison of arsenic concentration (μg/kg) in soil, root, and leaf/seed for wheat, tomato, and corn. Top three figure show plant species irrigated with wastewater (industrial area) and down three figure show plant species irrigated with river water (rural area).

The results showed that the soil, root, and seed data for arsenic concentration in wheat are not normal (*p <* 0.05). So, the Kruskal-Wallis nonparametric test was used. The results showed no significant differences among the groups in the soil, root, and seed. The highest concentration of arsenic was observed in the root (Fig. 5 and Table 3). Shapiro-Wilk test also showed that for tomato, the data of soil, root, and leaf are not normal (*p <* 0.05). The results showed significant differences among soil, root, and leaf (*p* = 0.026 for tomato and *p *= 0.00 for corn). Arsenic concentrations in the root were higher than other organs (soil = 43.67, root = 62.33, leaf = 32.67) (Fig. 5). In the case of rural area, the results showed a significant difference between arsenic concentrations of soil, root, and leaf/seed for wheat (*p* = 0.00) and tomato (*p *= 0.00), but there was no significant difference for corn (*p *= 0.125) (Fig. 5 and Table 3). Zhao et al. [5] investigated grain samples of 26 wheat cultivars grown in five field trials located in the productive farming regions in Europe. Grain from four trials contained low concentrations of total As (7.70 μg/kg), reflecting low levels of As in the soils (1.30–11 mg/kg). In contrast, at one of the trial sites, the As level in the soil was greater (29 mg/kg), and much higher As concentrations (69.00 μg/kg) were present in the wheat grain.

**Table 3:** The result of Kruskal-Wallis and ANOVA (Tukey) tests for the difference in arsenic concentration (μg/kg) among soil, root, and leaf/seed (wheat, tomato, and corn).

Industrial area	Wheat (Kruskal-Wallis)			Tomato (Kruskal-Wallis)			Corn (ANOVA (Tukey))		
	Groups	*p*	*Z*	Groups	*p*	*Z*	Groups	*p*	*F*
	Soil-Root	0.500	−0.0647	Soil-Root	0.046	−1.99	Soil-Root	0.043	119.56
	Soil-Seed	0.046	−1.99	Soil-Leaf	0.043	−2.02	Soil-Leaf	0.000	
	Seed-Root	0.046	−1.99	Leaf-Root	0.046	−1.99	Leaf-Root	0.000	

Rural area	Wheat (Kruskal-Wallis)			Tomato (Kruskal-Wallis)			Corn (ANOVA (Tukey))		
	Groups	*p*	*F*	Groups	*p*	*F*	Groups	*p*	*F*
	Soil-Root	0.002	12.00	Soil-Root	0.334	138.39	Soil-Root	0.483	3.00
	Soil-Seed	0.001		Soil-Leaf	0.000		Soil-Leaf	0.483	
	Seed-Root	0.000		Leaf-Root	0.000		Leaf-Root	0.109	

## Interspecies differences

**Fig. 6:**
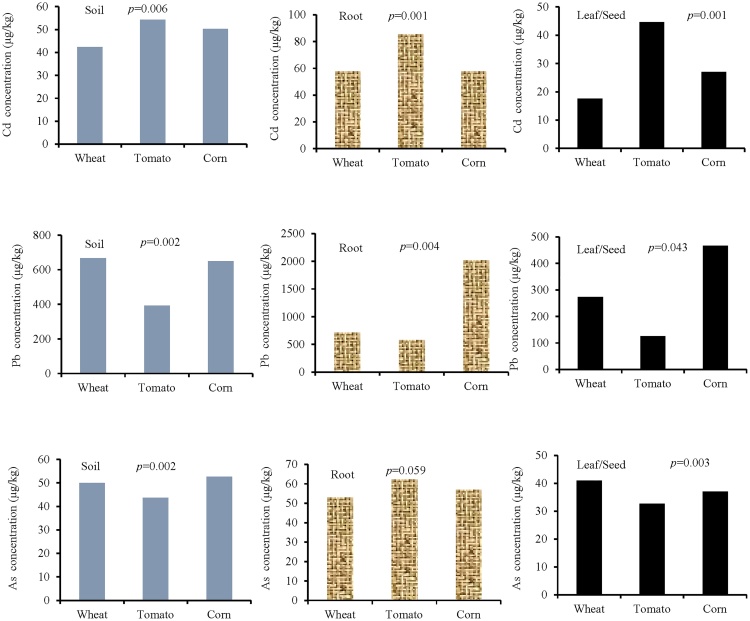
Statistical analysis of cadmium, lead, and arsenic concentrations (μg/kg) in soil, root, and leaf/seeds for wheat, tomato, and corn irrigated with wastewater (industrial area). These analyses have not been made for rural area.

In order to examine interspecies differences, the heavy metal concentrations between the three plants including wheat, tomato, and corn were compared (Fig. 6). This study showed that there is a significant difference in Cd concentrations among different plants in the soil, root, and leaf/seed in the industrial area (*p <* 0.05) except for As concentration in the root of the studied plants (*p* = 0.059). The mean soil Cd concentrations ranged from 42.33 (wheat) to 54.33 μg/kg (tomato). The mean root Cd concentrations ranged from 57.75 (wheat and corn) to 85.84 μg/kg (tomato). Furthermore, the mean leaf/seed Cd concentrations ranged from 17.66 (wheat) to 44.66 μg/kg (tomato). The results of this study showed that the concentration of Pb in the leaf/seed of wheat, tomato, and corn from the vicinity of industrial areas of Kashaf Rud, Mashhad were as 273.33, 126.66, and 466.66 μg/kg, respectively. Pb contents in the leaf of corn were significantly higher as compared to those in the other plants (*p* = 0.043). The results of the Farooq et al. [6] analysis showed that the concentration of Pb in the leaf of spinach, coriander, lettuce, radish, cabbage and cauliflower from the vicinity of industrial areas of Faisalabad, Pakistan were as 2.25, 2.65, 2.41, 2.03, 1.92 and 1.33 mg/kg, respectively. Pb contents in the leaves of coriander were significantly (*p <* 0.05) higher as compared to those in the other vegetables, whereas, the leaf samples of cauliflower were found to be significantly (*p *< 0.05) lower in Pb contents. However, there was no significant (*p *> 0.05) variation in the level of Pb in the leaves of spinach and lettuce. Stems of spinach, coriander, lettuce, radish, cabbage, and cauliflower contained 1.19, 1.64, 1.88, 2.16, 1.62 and 1.31 mg/kg, respectively. Higher levels of Pb were determined in radish stems, whereas considerably (*p *< 0.05) lower amount of Pb was found in spinach stems, but no considerable difference of Pb concentration was observed in the leaves of coriander and cabbage (*p *> 0.05). The roots of spinach, coriander, lettuce, radish, cabbage and cauliflower contained 1121, 1.53, 1.85, 2.25, 1.15 and 1.22 mg/kg of Pb contents respectively. In this study, the results of Tukey test showed that there was a significant difference in As concentrations among different plants (*p *= 0.002 for soil and *p* = 0.003 for leaf/seed). The highest arsenic concentration was in the root of tomato (62.33 μg/kg). The root of tomato ant soil of corn had intermediate values, whereas, the soil of tomato and leaves of wheat were in subsequent orders, and the lowest concentration of As was in leaves of tomato (32.67 μg/kg). As absorption by plants is influenced by many factors including plant species [7], As concentrations and forms in the soil [8], soil properties such as pH and clay content [9], and the presence of other ions [10]. Most plants grown on As-contaminated soils contain elevated As levels. For most plants, the highest concentrations of As are found in roots, with the aboveground vegetative parts (leaves and stems) being lower, and the lowest levels found in fruit and seeds [11].

## Differences between industrial and rural areas

**Fig. 7:**
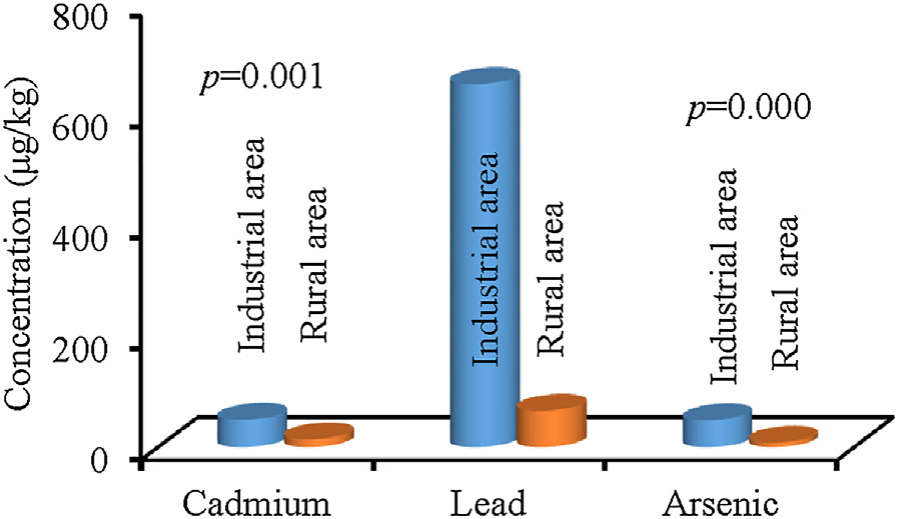
Statistical analysis of cadmium, lead, and arsenic concentrations (μg/kg) between industrial area and rural area.

The heavy metal concentrations between industrial and rural areas were compared (Fig. 7). Demirezen and Ahmet [12] analyzed different samples of vegetables and reported a high concentration (3.00–10.70 mg/kg) of Pb which poses health risks to human life. In another study, Sharma et al. [13] investigated that concentration of Pb in vegetables grown in industrial areas (17.54–25.00 mg/kg). Muchuweti et al. [14] reported the level of Pb in vegetables irrigated with mixtures of wastewater and sewage from Zimbabwe (6.77 mg/kg). Fytianos et al. [15] examined a high concentration of Pb in spinach grown in industrial and rural areas of Greece. Sewage effluents are considered not only a rich source of organic matter and other nutrients but also they elevate the level of metals like Cd, As, Fe, Mn, Cu, Zn, Pb, Cr, Ni, and Co in receiving soils [16].

## Heavy metal standards

The mean concentrations of cadmium, lead, and arsenic in the samples were compared with the standards of United State Environmental Protection Agency (USEPA), United Kingdom (UK), Union of Europe (EU), World Health Organization (WHO) [17], Iran Department of Environment (DOE) and etc. (Table 4). The amount of Cd in the soil samples of different plants including *Triticum aestivum* L., *Zea mize* and *Solanum lycopersicum* was found to be as 42.33, 50.33, and 54.33 μg/kg, respectively. The results of soil quality monitoring carried out by the Poland Ministry of Agriculture and Rural Development [18] in 2000 showed that the lowest cadmium concentration, about 0.04 mg/kg, was recorded in the northeastern Poland (including Warmia and Mazury) and central Poland [19]. According to the Regulation on Soil Quality Standards of 2002 by the Poland Minister of Environmental Protection [20], the permissible cadmium concentration is much higher, e.g. 1 mg/kg in the protected areas, and 4 mg/kg in the surface layer of arable soils.

**Table 4:** Maximum admissible concentrations of toxic metals (mg/kg) in soil and plant from the international and national standards.

Reference	Samples form	Heavy metals standards (mg/kg)		
		Cadmium	Lead	Arsenic
United Kingdom	Soil	3.00	300	20.0
Union of Europe	Soil	1.00–3.00	50–300	–
	Plant	0.20	–	–
Environmental Protection Agency	Soil	–	40.0	–
World Health Organization	Soil	0.20	–	–
	Plant	–	0.30	0.70
Iran Department of Environment	Soil	5.00	75.0	40.0
Canada	Soil	–	–	25.0
Japan	Soil	–	–	15.0
Poland Minister of Environmental Protection	Soil	4.00	100	30.0
Poland Minister of Agriculture and Rural Development	Soil	0.75–1.5	50–100	–
Poland Minister of Health	Plant	0.1	0.2	–

Heavy metal accumulation in agricultural soils can be a problem when using wastewater to irrigate the soil. Increased levels of heavy metals in the soil, strongly affect the pH, Cation Exchange Capacity (CEC), organic matter, soil mineral elements, dynamics and mobility of soil [21]. The effect of heavy metals accumulation in soil depends on many factors such as the concentration of heavy metals in the sewage, irrigation duration, pH, soil texture, and CEC. In fact, after 10–50 years, the levels of heavy metals exceed the standards in the soils irrigated with wastewater [22]. The results of Rattan et al. [23] showed that the use of wastewater in irrigation for 10 years increases iron, nickel, and lead concentrations in the soil.

The large agricultural fields are irrigated by the untreated industrial and domestic wastewater in Iran. This type of irrigation with polluted water causes the low quality of agricultural products and reduction of soil productivity. On the other hand, an increase of the heavy metal concentration in the soil as high as the toxic level irrigated with polluted water may reach to the human and animal food as like affect the plant growth and quality. In the case of water used in watering plants, the maximum concentration of cadmium proposed is equal to 0.01 mg/L. In concentrations of 0.1 mg/L, nutritious solutions for beans, beets, and turnips are toxic. Standard of cadmium in drinking water is 0.005 mg/L. The proposed maximum concentration of lead in the water used for watering plants is 5 mg/L. Lead standard in drinking water is 0.05 mg/L. High concentrations of heavy metals in the sewage with long-term use of wastewater in irrigation can cause a significant increase in the soil heavy metals [24]

Table 4 shows the standard of heavy metals studied in plants. The lead concentration in the seeds of wheat was 273.33 μg/kg. According to the Regulation by the Poland Minister of Health [25], the calculated mean lead concentration in the wheat seed is higher than the maximum acceptable level (200 μg/kg). In Standard Methods for the Examination of Water and Wastewater, the allowable level of lead in plants for human consumption is cited as 2 ppm [26]. Lead contamination in plants seeks to prevent root elongation and photosynthesis. The sensitivity and response of plants to lead are different and depend on the physiological and genetic structure. The lead in soil is absorbed by plants, which is stored mainly in the roots. The allowable concentration of arsenic in plants recommended by the World Health Organization is 0.7 mg/kg dry weight, while the concentrations found in the studied plants are much lower. Nazemi et al. [27] examined arsenic, chromium, cadmium, lead and zinc in vegetables grown in Shahrud, Semnan province and concluded that the mean concentrations of heavy metals are more than the Food and Agriculture Organization (FAO) levels. Lead concentrations in plants studied were more than the reference value reported by the American Water Works Association (100 μg/kg).

## Quantitative risk assessment

Vegetables, wheat, and rice constitute the major part of the diet of the Iranian people. In this study, it was assumed that the local population consume the local wheat and the ADD_pot_ calculated from Eq. (2) is based on heavy metal levels from the seeds of wheat samples (concentrations of toxic material in the industrial area = 17.66, 273.33, and 41.66 μg/kg for cadmium, lead, and arsenic, respectively). The intake of elements in wheat consumption was calculated daily [28]. The total daily intake (ADD_pot_) of cadmium, lead, and arsenic in the industrial area was as 0.07, 1.17 and 0.17 μg/kg/day, respectively. Furthermore, the total daily intake (ADD_pot_) of cadmium, lead, and arsenic in the rural area was as 0.04, 0.05 and 0.00 μg/kg/day, respectively. Tolerable daily intake (Provisional Tolerable Daily Intake: PTDI) according to US Environmental Protection Agency standard for cadmium, lead, and arsenic is equal to 1.00, 3.75, and 2.14 μg/kg/day, respectively [29]. Therefore, the ADD_pot_ for all elements was less than the PTDI. The RfD values are as 1.00, 14.00, and 0.30 μg/kg/day for cadmium, lead, and arsenic, respectively. Risk potential (THQ) of each element for wheat consumption in Kashaf Rud, the industrial area, was calculated for the individual consumer. The risk potential of wheat consumption was 0.07 for cadmium, 0.08 for lead and 0.56 for arsenic. Furthermore, the risk potential of each element for wheat consumption in Sar Rud, rural area, was calculated for the individual consumer. The risk potential of wheat consumption was 0.003 for lead, 0.04 for cadmium, and 0.00 for arsenic. The results of this study indicated that among the elements, As was more dangerous than others. Huang et al. [30] investigated the risk assessment of heavy metals on human health through consumption of rice in the state of Changshu in East China, which showed amounts of THQ for rice as Zn < Cu < Cr < As < Cd < Hg < Pb. Chary et al. [31] investigated the risk of heavy metals in vegetables grown in lands irrigated with wastewater, which showed that the risk for zinc, lead, and chromium is high.

The LADD_pot_ from Eq. (3) was calculated as 0.17 and 0.00 μg/kg./day in the industrial and rural areas, respectively. The cancer slope factor for arsenic values is 1.5 mg/kg/day. The cancer risk of arsenic for wheat consumption in Kashaf Rud was calculated for the individual consumer. The risk potential of wheat consumption was equal to 255 × 10^−6^ and 0.00 in industrial and rural areas, respectively (from Eq. (5)). The cancer risk for the industrial area is greater than 1 × 10^−6^, which is unacceptable. Chamannejadian et al. [32] reported that the ADD_pot_ of Cd for rice consumption by the local population was as 0.59 μg/kg/day. This value corresponds to 59% of the PTDI (1 μg/kg/day). The maximum daily intake of Cd from rice was 1.13 μg/kg/day, which calculated from the maximum content rations of Cd in rice in Ahvaz, and was 0.13-fold greater than the PTDI. The study of Khani and Malekoti [33], showed that averaged Cd content in the rice produced in the north of Iran was 0.34 mg/kg with a range of 0.25–0.45 mg/kg. It is important to note that the calculated ADD_pot_ in this study for Cd, Pb, and As (which are less than PTDI) were only obtained through wheat consumption, and the heavy metal intake through dietary would probably increase the ADD_pot_ values. Some researchers also reported similar amounts of ADD_pot_ of Cd and Pb through rice consumption [34]. Investigation of Cd content of rice from different countries revealed a range of 0.0008–0.13 mg/kg with the average of 0.03 mg/kg. The mean Cd content values in rice seeds reported for Japan were 50 ng/kg dry wt in 1998–2000 and 0.01 mg/kg dry wt for Taiwan in 2004 [35].

## Additional information

Among the factors disturbing the ecosystem, some are more important like pollutants and heavy metals due to their physiological effects on living organisms at low concentrations [[36], [37], [38]]. Monitoring of heavy metals in the environment is essential to identify the current state of the environment. In this path, we can predict the future of the environment [[39], [40], [41], [42]] (. Some studies have been done all over the world in relation to plants and soil contamination with heavy metals from urban and industrial effluents [43]). Lead (Pb), as the most widespread heavy metal in the environment, affects the metabolic and physiological activities of living organisms. In general, most scientists believe that the lead contamination is the most serious form of metal pollution, which has a significant impact on human health. Pb is known to induce renal tumors, reduce cognitive development, and increase blood pressure in adults. Other symptoms of Pb toxicity include gastrointestinal disorders and some liver impairment. Other metals such as Cadmium (Cd) are toxic even at low concentrations, which are not known to have any important biological properties in the humans [44]. Cd may induce osteomalacia and reproductive deficiencies. It can also cause damage to the central nervous system and produce psychological disorders. In fact, there is evidence that prolonged exposure to cadmium ultimately leads to an increased risk of kidney disease. Arsenic (As) is another element that is widely distributed in the Earth's crust [45]. Arsenic in the food system is a topic of much discussion, with particular attention focused on it over the past year through reports on levels in the juices and rice. The most commonly cited consequences of chronic exposure to low levels of inorganic arsenic include increased incidence of bladder, lung, kidney and skin cancers, the elevated levels of heart disease, skin hyperpigmentation, and skin lesions. All researches published to date links the occurrence of these health impacts to chronic exposure through contaminated water sources. Arsenic can be found in the processing industries of copper, lead, gold and other non-ferrous metals, glass, microelectronics, wood preservation, semiconductor detectors, batteries, dyes, taxidermy and tanning waste from nuclear power plants, fossil fuel burners, veterinary medicines and food additives [46]. The first international standard for an acceptable level of arsenic in drinking water has been as 0.2 mg/L. In 1963, the drinking water standard was revised and reduced the arsenic levels to 0.05 mg/L. Water is the most studied area for inorganic arsenic reduction and elimination, and existing technology can effectively reduce whatever existing levels are present in drinking water to below the 10 ppb standard established by the U.S. Environmental Protection Agency (EPA) for drinking water, and by Food and Drug Administration (FDA) for bottled water. It is recommended when the amount of arsenic in water is more than 0.05 mg/L, the first step should be determining the capacity and chemical forms. The average concentration of arsenic in the unpolluted soil is between 5 and 10 μg/g [45]. The emission of anthropogenic heavy metal compounds causes considerable changes in biogeochemical cycles of some elements. Following their uptake from contaminated soils by food plants, heavy metals are included in the human food chain. Furthermore, heavy metal accumulation in plants evokes their stress responses. The contamination of agricultural soils is often a direct or indirect consequence of anthropogenic activities. Sources of anthropogenic metal contamination in soils include urban and industrial wastes; mining and smelting of non ferrous metals and metallurgical industries [47] Disposal of sewage water and industrial wastes is a great problem. It is often drained to the agricultural lands, where it is used for growing crops, including vegetables [16]. As a result, it leads to contamination of the food chain, because vegetables absorb heavy metals from the polluted soil. Estimated daily intake, as a common index for metal transfer from plant to humans, was calculated and used for rice in some studies [32].

This paper provides the first quantitative information on accumulation of heavy metals (lead, cadmium, and arsenic) in ground (soil), overground (leaf), and underground (root) parts of wheat (*Triticum aestivum* L.), corn (*Zea Maize*), and tomato *(Solanum lycopersicum*) in the downstream agricultural lands of an industrial area (Razavi Khorasan province, Mashhad city, Khin Arab area, Kashaf Rud River Basin) and agricultural lands of a rural area (Razavi Khorasan province, Kalat city, Sar Rud village). In addition, health risk assessment of consumers was done by EPA/WHO instructions in this multispecies monitoring. The main objective was to evaluate the potential health risks associated with heavy metals via consumption of wheat from the area using the Average Daily Dose for Intake Process (ADD_pot_) and Hazard Quotient (HQ) from heavy metals. Finally, the cancer risk of arsenic for consumption of wheat seed was calculated.

A human health risk assessment is the process to estimate the nature and probability of adverse health effects in humans who may be exposed to chemicals in contaminated environmental media, now or in the future. Four steps to a health risk assessment document are including [2]:

### Hazard identification

Hazard identification involves gathering and evaluating toxicity data on the types of health injury or disease that may be produced by a chemical and the conditions of exposure under which injury or disease is produced. The subset of chemicals selected for the study is termed “chemicals of potential concern”.

### Dose-response assessment

The dose-response assessment involves describing the quantitative relationship between the amount of exposure to a chemical and the extent of toxic injury or disease. The US EPA established the Reference Dose (RfD, μg/kg/day) for dose-response assessment.

### Exposure assessment

Exposure assessment applies a generalized dose-response relationship to specific conditions for some population. Exposure assessment involves describing the nature and size of various populations exposed to a chemical agent, and the magnitude and duration of their exposures. The exposure pathway of heavy metals to human through ingestion of contaminated food has been studied by many researchers. Potential Average Daily Dose for intake process (ADD_pot_, μg/kg/day) is calculated for exposure assessment. For effects such as cancer, where the biological response is usually described in terms of lifetime probabilities, even though exposure does not occur over the entire lifetime, doses are often presented as lifetime average daily doses (LADDs).

### Risk calculation

For risk calculation, the Average Daily Dose for Intake Process (ADD_pot_) (total intake) is compared to the RfD. The result of this comparison is Hazard Quotient (HQ) [48]. Using cancer slope factor and exposure data in μg/kg/day, cancer risks are calculated.

In this study, lead concentrations in corn leaves (466.66 μg/kg) were more than the reference value reported by WHO. The daily intake of lead, cadmium, and arsenic uptake rate was lower than the tolerable daily (PTDI) determined by FAO/WHO. The results of this study suggest that the risk of noncancerous diseases of wheat consumption is not high for consumers. Furthermore, the results of this study indicated that among the elements, arsenic was more dangerous than others (Risk potential (THQ) = 0.56). The cancer risk of arsenic in wheat for the industrial area is greater than 1 × 10^−6^, which is unacceptable. This study does not cover all areas. It seems that doing further research is necessary. However, the following suggestions for environmental management are noteworthy:

1 Annual monitoring and measuring the amounts of heavy metals in the samples analyzed and the major vegetables
2 The annual monitoring and measurement of heavy metals in soil of farmland and production of a database
3 Increasing public awareness to local residents about the using wastewater
4 Increasing the wastewater treatment plant modules
5 A permanent control of the meat and milk of animals and crops grown with sewage
6 Investigation of daily intake of heavy metals in the diet of whole grains and vegetables in Mashhad
7 Calculation of heavy metals potential risk in whole grains and vegetables in Mashhad.
